# A broad spectrum, one-step reverse-transcription PCR amplification of the neuraminidase gene from multiple subtypes of influenza A virus

**DOI:** 10.1186/1743-422X-5-77

**Published:** 2008-07-09

**Authors:** Alejandra Castillo Alvarez, Marion EG Brunck, Victoria Boyd, Richard Lai, Elena Virtue, Wenbin Chen, Cheryl Bletchly, Hans G Heine, Ross Barnard

**Affiliations:** 1 Biochip Innovations Pty Ltd., 8 Mile Plains, Queensland, Australia; 2 CSIRO livestock Industries, Australian Animal Health Laboratory (AAHL), Geelong, Vic, Australia; 3 Australian Biosecurity Cooperative Research Centre for Emerging Infectious Disease, The University of Queensland, St. Lucia, Queensland, Australia; 4 Pathology Queensland, Central Laboratory, Herston Hospitals Campus, Herston, Queensland, Australia; 5 School of Molecular and Microbial Sciences, The University of Queensland, St. Lucia, Queensland, Australia

## Abstract

**Background:**

The emergence of high pathogenicity strains of Influenza A virus in a variety of human and animal hosts, with wide geographic distribution, has highlighted the importance of rapid identification and subtyping of the virus for outbreak management and treatment. Type A virus can be classified into subtypes according to the viral envelope glycoproteins, hemagglutinin and neuraminidase. Here we review the existing specificity and amplification of published primers to subtype neuraminidase genes and describe a new broad spectrum primer pair that can detect all 9 neuraminidase subtypes.

**Results:**

Bioinformatic analysis of 3,337 full-length influenza A neuraminidase segments in the NCBI database revealed semi-conserved regions not previously targeted by primers. Two degenerate primers with M13 tags, NA8F-M13 and NA10R-M13 were designed from these regions and used to generate a 253 bp cDNA product. One-step RT-PCR testing was successful in 31/32 (97%) cases using a touchdown protocol with RNA from over 32 different cultured influenza A virus strains representing the 9 neuraminidase subtypes. Frozen blinded clinical nasopharyngeal aspirates were also assayed and were mostly of subtype N2. The region amplified was direct sequenced and then used in database searches to confirm the identity of the template RNA. The RT-PCR fragment generated includes one of the mutation sites related to oseltamivir resistance, H274Y.

**Conclusion:**

Our one-step RT-PCR assay followed by sequencing is a rapid, accurate, and specific method for detection and subtyping of different neuraminidase subtypes from a range of host species and from different geographical locations.

## Background

Influenza viruses are enveloped, segmented, negative sense RNA viruses of the family *Orthomyxoviridae *and are classified into types (A, B or C) based on the antigenic difference in their nucleoproteins (NP) and matrix proteins (M1). Influenza virus A and B infections are an important cause of morbidity and mortality in humans and in a wide range of animal species [1-3]. Type "A" viruses are the most important pathogens of the three types and have been associated with all of the past influenza pandemics [4,5].

Influenza A viruses are classified into subtypes according to the hemagglutinin (HA) and neuraminidase (NA) glycoproteins that are present in the viral envelope. There are 16 subtypes of HA (H1-H16) and nine subtypes of NA (N1-N9) identified by serology [6]. Different combinations of HA and NA subtypes are found in wild birds which are the natural reservoir of influenza viruses. In contrast, only some subtypes are commonly found in humans. For instance, three HA subtypes and two NA subtypes have established stable lineages in humans and have been routinely isolated since last century (e.g. H1N1 in 1918, H2N2 in 1957, and H3N2 in 1968) [7-9]. However, a number of viruses have reemerged over recent years and various subtypes of influenza A virus including H5N1, H9N2, H7N7, H7N3, and H7N2 have been reported to infect humans [10,11]. Most importantly, recent outbreaks of highly pathogenic H5N1 in different continents have shown that the virus jumps the species barrier from poultry to humans, causing high mortality in both species, even though the virus is not easily transmitted from humans to humans [10,12,13]. These findings reiterate the importance of influenza virus surveillance in poultry and humans.

In addition, the latest outbreak of influenza A in horses in Australia also highlights the importance of animal surveillance during and post outbreak. Equine influenza is considered the most economically important respiratory disease of horses in countries with significant racing and breeding industries, with subtype H3N8 the predominant subtype [14].

Rapid and accurate subtyping of influenza A virus is crucial for the diagnosis and surveillance of emerging viruses and for outbreak management, as well as for determining the appropriate treatment and presence of drug resistant strains.

Traditionally, the gold standard for virus detection involves virus replication in eggs or tissue culture followed by HA inhibition [15,16] and NA inhibition assays [17,18]. However, these inhibition assays are laborious, not very sensitive and do not provide results in a period that allows for optimal use of potentially effective antiviral treatment [14,19,20]. To date, influenza diagnostic methods based on reverse transcription-PCR (RT-PCR) and real-time RT-PCR (RRT-PCR) are currently available for HA, but they are not well developed for NA. Current NA PCR tests only identify a few subtypes (e.g. N1-N2) [21-23]. The only assay identifying all 9 NA subtypes is a nested two-step RT-PCR method followed by cloning and sequencing described by Hoffman *et al*.[24]. There is no published data on how the former method behaves (ie. sensitivity and specificity) when clinical samples are assayed, which might represent a problem when amplifying a full length NA gene (1.5 kb) in these type of samples. The whole process for subtyping is slow and prone to contamination (because it is a nested PCR), which might not be a practical test for routine surveillance work or post-diagnostic studies.

Therefore, there is a need for relatively simple and fast test that provides subtype and sequence information of all known NA subtypes, targeting a spectrum of hosts with acceptable sensitivity. To this end, we have designed a primer set for a one-step RT-PCR detection assay of multiple influenza A viruses, based on the detection of the NA gene. Our one-step RT-PCR method, followed by sequencing, was validated with a panel of 32 allantoic fluids containing influenza A viruses, representing the 9 NA subtypes obtained from a range of host species and from different geographic locations. Archived clinical specimens, mainly from Queensland, Australia were also tested. Efficacy of our method was compared with the traditional neuraminidase inhibition test [17,18]. From our findings, we concluded that this one-step RT-PCR assay followed by sequencing is a rapid and specific method for influenza A virus detection and NA subtyping.

## Results

### Design of oligonucleotides

Despite the high sequence variability of the NA gene between subtypes of influenza "A" viruses, we used entropy plots (data not shown) to identify semi-conserved regions, where primers could be designed. Two target regions that potentially identify all possible NA subtypes were chosen. Primer NA8F corresponds to the region from base 690 to 708 and primer NA10R to the region from base 890 to 909 (base numbering corresponds to reference sequence Genbank accession number DQ139321). The two primers without M13 tags amplified an approximate 219 bp NA fragment which includes one mutation site known to be related to oseltamivir resistance (e.g. virus subtype N1: amino acid H274Y). There are other residues that confer resistance to the NA inhibitors, but for the purpose of this study we focused on the H274Y mutation (an NA mutation that appears to be increasing significantly in frequency and distribution [25]).

**Figure 1:**
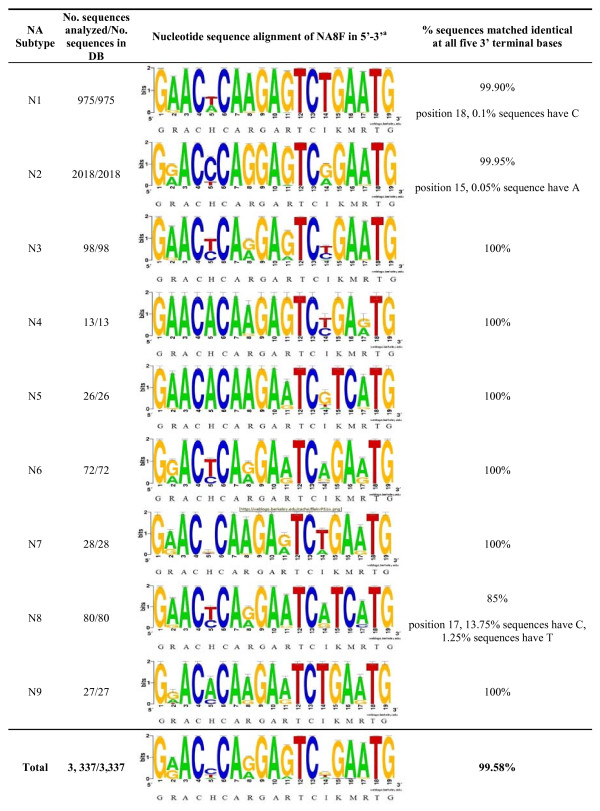
**Nucleotide sequence alignment of NA8F primer**. A sequence logo representation of 3,337 available sequences in the NCBI database at the time the study was conducted. All 9 NA subtypes were aligned against the NA8F primer and analyzed for discrepancies at the 3'end. The big letters represent the consensus sequences for each subtype. The standard mixed base definition was applied, and for reference "I" stands for inosine. ^a^Alignment is presented in sequence logo format [32-34].

**Figure 2:**
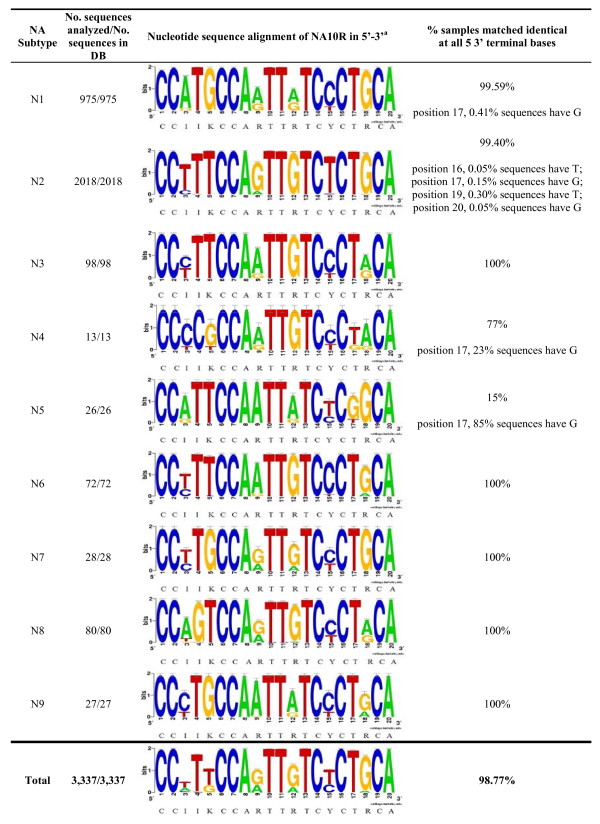
**Nucleotide sequence alignment of NA10R primer**. A sequence logo representation of 3,337 available sequences in the NCBI database at the time the study was conducted. All 9 NA subtypes were aligned against the NA10R primer and analyzed for discrepancies at the 3'end. The big letters represent the general consensus sequences for each subtype and at the end a general consensus sequence is given for the total population of samples. The standard mixed base definition was applied, and for reference "I" stands for inosine. ^a^Alignment is presented in sequence logo format [32-34].

**Figure 3:**
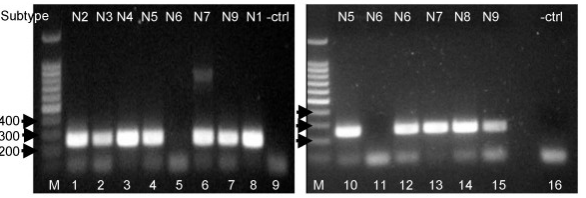
**One-step RT-PCR amplification of NA gene from all 9 NA subtypes using animal samples from allantoic fluids**. A fragment of approximately 253 bp was amplified using primers containing M13 sequence. Example of some subtypes (refer to Table 2 for strain names) assayed: M, 100 bp DNA Ladder (Promega); 1) H9N2, 2) H16N3, 3) H8N4, 4) H14N5, 5) H13N6, 6) H10N7, 7) H11N9, 8) H5N1, 9) negative control (water instead of template), 10) H6N5, 11) H13N6, 12) H14N6, 13) H7N7, 14) H3N8, 15) H11N9, 16) negative control (water instead of template).

Bioinformatic analysis of each primer is presented in sequence logo format as shown in Figures 1, 2. The NA8F and NA10R primers were aligned against 3,337 sequences in the NCBI IVRD. When analyzing the last five bases at the 3'end of the primer NA8F, the alignment gave close to 100% match for all subtypes. In the case of the primer NA10R, the alignment gave close to 100% predicted match in the last 5 bases for all subtypes except for N2, N4, and N5 where it was 99.40%, 77% and 15% respectively. The N2 subtype has variability in the last five bases, but the frequency of variability is so low such that there is only one nucleotide difference in any single mismatched virus sequence. Despite the percentage of predicted mismatch in the last five 3' terminal bases of N4 and N5 subtypes they are detected with our primers. Thus, all 9 NA subtypes can be amplified as shown in Table 2 and Figure 3.

**Table 1:** Identification of neuraminidase subtypes of influenza A clinical nasopharyngeal aspirates.

**Sample ** No. *	**HA ** **subtype^a^**	**NA ** **subtype**	**Subtype**	**Amplification by ** **our RT-PCR**	**sequence**
1	H3	NVI^1^	H3	-	-
2	H3	N2	H3N2 Wisconsin/67/2005 like strain	+	N2
3	H3	NVI^1^, N/A^2^	H3	+	N2
5	H3	N2	H3N2 Wisconsin/67/2005 like strain	+	-
6	H3	N2	H3N2 Wisconsin/67/2005 like strain	+	N2
8	H3	N2	H3N2	+	N2
9	H3	NVI^1^, N/A^2^	H3	-	-
10	H3	N2	H3N2	+	N2
12^e^	H1	N1	H1N1	+	N2
14	H3	N2	H3N2 Wisconsin/67/2005 like strain	-	-
15	H3	N2	H3N2 Wisconsin/67/2005 like strain	+	N2
17	H3	NVI^1^, N/A^2^	H3	+	NSA^3^
19	H3	N2	H3N2 Wisconsin/67/2005 like strain	+	N2
21	H3	NVI^1^, N/A^2^	H3	+	N2
26	H3	N2	H3N2 Wisconsin/67/2005 like strain	-	-
27	H3	NVI^1^	H3	-	-
30	H3	N2	H3N2 Wisconsin/67/2005 like strain	+	N2
31	H3	NVI^1^	H3	+	NSA^3^
32	H3	N2	H3N2 Wisconsin/67/2005 like strain	+	N2
33	H3	N2	H3N2 Wisconsin/67/2005 like strain	+	N2
34	H3	N2	H3N2 Wisconsin/67/2005 like strain	+	N2
36	H3	NVI^1^	H3	-	-
37	H3	N2	H3N2 Wisconsin/67/2005 like strain	+	N2
38	H3	N2	H3N2 Wisconsin/67/2005 like strain	+	N2
42	H3	N2	H3N2 Wisconsin/67/2005 like strain	+	N2
45	H3	N2	H3N2 Wisconsin/67/2005 like strain	+	N2
47	H3	NVI^1^, N/A^2^	H3	-	-
50	H3	NVI^1^, N/A^2^	H3	-	-
51	H3	N2	H3N2 Wisconsin/67/2005 like strain	+	N2
54	H3	N2	H3N2 Wisconsin/67/2005 like strain	+	N2
55	H3	N2	H3N2 Wisconsin/67/2005 like strain	+	N2
56	H3	NVI^1^, N/A^2^	H3	-	-
57^c^	NVI^1^	NVI^1^, N/A^2^	Not available	-	-
62^d^	H5	N/A^2^	H5	+	N2
63^d^	H5	N/A^2^	N/A^2^	-	-
64^d^	Not H5	N/A^2^	N/A^2^	-	-
65	H3	N2	H3N2 Wisconsin/67/2005 like strain	+	N2
67^f^	-	N/A^2^	Not available	+	NSA^3^

*Blinded specimens provided by Pathology Queensland. Only typed A influenza samples were listed. Type B influenza samples, adenovirus, and RSV samples were not included as they were all negative by our assay.

^a ^HA subtyping was performed by Queensland Forensic and Scientific Services using real-time PCR based on HA specific primers, as well as by hemaglutination and inhibition test to the viruses that were able to be cultured.

^c ^Sample 57 was not confirmed by a second laboratory for Influenza A infection nor was there virus isolated.

^d ^Samples 62, 63 and 64 are not clinical samples but are positive control RNA sample from WHO reference lab.

^e ^Sample was assayed and subtyped in duplicate to corroborate the NA subtype by sequence.

^f ^Sample 67 had pink contaminants floating in the RNA that could have affected PCR result.

NVI^1 ^= No virus isolated, therefore NA subtyping was unfeasible.

N/A^2 ^= Sample was not assayed with the corresponding test.

NSA^3 ^= RT-PCR was positive but we were not able to obtain subtype by direct sequencing.

### Detection of influenza A virus by one-step RT-PCR and NA subtyping

**Figure 4:**
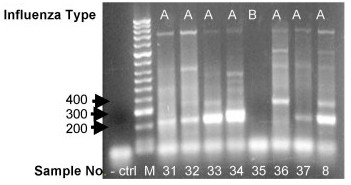
**One-step RT-PCR amplification of NA gene from clinical NPA samples**. Example of amplification results of Influenza A and influenza B. Sample numbers correspond to the samples described in Table 1, except sample 35 which is influenza B (description data not shown); M, 100 bp DNA Hyperladder II (Bioline); and negative control (- ctrl: water instead of template).

Freeze-thawed blinded clinical NPA (see "clinical samples", later revealed to consist of a mix of influenza A, influenza B and adenovirus) were assayed by our one-step RT-PCR using NA8F/NA10R primers followed by indirect sequencing. Our clinical data showed amplification of the expected 219 bp NA fragment from 25/37 (68%) of freeze stored influenza A samples and these were mostly N2 subtype (see Table 1 and Figure 4). From these influenza A clinical NPAs samples, two of them labeled as #17 and # 31 in Table 1 were initially amplified by our primers but did not re-amplify using NA M13 tagged primers. We could not repeat the assay because our RNA stock of those 2 samples was exhausted. The fact that there was no virus isolated for those samples does not necessarily explain the lack of amplification, as we clearly amplified and subtyped clinical NPAs samples #3 and #21 which also had no virus isolated.

In addition to the influenza A clinical samples tested, 30 blinded negative controls were assayed: 24/25 samples classsifed as influenza B by a hospital pathology service were negative and 1/25 was positive; 1/1 adenovirus sample and 4/4 RSV samples were negative. It is unclear why the one sample labeled as influenza B was positive (clinical NPA # 67), however, there was evidence of macroscopic contaminants in this sample (colored particulates), leading us to question its integrity. We were unable to reproduce the amplification of the later sample for further sequence analysis.

In order to speed the procedure for routine post-diagnostic application, M13 extensions or "tags" were added to the primers, which, as a serendipitous benefit, reduced non-specific banding in some of the samples previously assayed with NA8F/NA10R primer set (data not shown). Therefore, to validate our one-step RT-PCR method using M13 tagged primers followed by direct sequencing, a panel of influenza A virus strains (in allantoic fluids) representing the 9 NA subtypes was assayed (see Table 2). In total, 31/32 (97%) were amplified and the subtyping results were compared with the traditional neuraminidase inhibition assay. The identity of the 253 bp fragments was confirmed by direct sequence analysis and BLAST search (see Table 2). The sample that did not amplify was influenza A/Gull/Maryland/704/77/H13N6 (Fig. 3, lanes 5 and 11); however the other H13N6 sample influenza/A/Gull/Tas/06 (not shown) did amplify, as did the other eight N6 subtypes including A/Mallard/Gurjev/244/82 (Figure 3, lane 12). It is unclear why the amplification of one N6 sample failed, because none of the N6 subtype full length sequences from the IVRD database (n = 119), including the ones for that particular H13N6 (Genbank accession numbers AY207553 and CY014696), have destabilizing mutations in the primer annealing sites.

**Table 2:** Identification of all neuraminidase subtypes by RT-PCR followed by direct sequencing using animal samples from allantoic fluids.

**Influenza virus strain (^a^)**	**Subtype (^b^)**	**One-step RT-** **PCR (^c^)**	**NA subtype by ** **sequence (^d,e^)**
1) A/Chicken/Vietnam/8/04	H5N1	+	N1
2) A/Chicken/Laos/26/06	H5N1	+ (8)	N1
3) A/Chicken/Cambodia/1A/04	H5N1	+	N1
4) A/Chicken/Malacca/4905/03	H9N2	+ (1)	N2
5) A/Gull/Denmark/68110/02	H16N3	+ (2)	N3
6) A/Shearwater/Aust/75	H5N3	+	N3
7) A/Grey teal/WA/1762/79	H4N4	+	N4
8) A/Emu/NSW/97	H7N4	+	N4
9) A/Turkey/Ontario/6118/67	H8N4	+ (3)	N4
10) A/Shearwater/Aust/72	H6N5	+ (10)	N5
11) A/Mallard/Gurjev/263/82	H14N5	+ (4)	N5
12) A/Mallard/Gurjev/244/82	H14N6	+ (12)	N6
13) A/Gull/Maryland/704/77	H13N6	Neg (5, 11)	Not typed
14) A/Gull/Tas/06	H13N6	+	N6
15) A/Duck/NZ/89	H4N6	+	N6
16) A/Grey teal/WA/1855	H4N6	+	N6
17) A/Duck/Viet/317/2005	H4N6	+	N6
18) A/Duck/Viet/318/2005	H4N6	+	N6
19) A/Duck/Viet/323/2005	H4N6	+	N6
20) A/Duck/Viet/342/2005	H3N6	+	N6
21) A/Duck/Vic/512/2007	H7N6	+	N6
22) A/Duck/Victoria/1/76	H7N7	+ (13)	N7
23) A/Chicken/Germany/N/49	H10N7	+ (6)	N7
24) A/Chicken/Victoria/1/85(^f^)	H7N7	+	N7
25) A/N. Korean	H7N7	+	N7
26) A/Avian/669/WA/78	H3N8	+ (14)	N8
27) A/Equine/Sydney/2888-8/2007	H3/N8	+	N8
28) A/Tern/Aust/75	H11N9	+ (7, 15)	N9
29) A/Shelduck/WA/1757/78(^f^)	H1N9	+	N9
30) A/Red-necked stint/WA/5745/84(^f^)	H12N9	+	N9
31) A/Shelduck/WA/1762/79	H15N9	+	N9
32) A/Wedge tailed shearwater/WA/2327/1983	H15N9	+	N9

(^a^) Strains from the collection at the Australian Animal Health Laboratory were kindly provided by Paul Selleck. RNA was extracted from allantoic fluid.

(^b^) Subtypes of virus stock were determined previously at AAHL by HA and NA inhibition assays according to Barr and O'Rourke [38], Van Dusen, *et al*. [18]and Aymard-Henry, *et al*. [17].

(^c^) RT-PCR fragment visualized in agarose gel electrophoresis. In brackets refer to lane numbers on agarose gel, Figure 3; not all results shown on gel)

(^d^) Sequencing directly from gel purified one-step RT-PCR using M13 tagged primers.

(^e^) Results of Blastn analysis.

(^f^) These samples were assayed by two-step RT-PCR before transferring to one-step RT-PCR.

### Sensitivity of the one-step RT-PCR assay

**Figure 5:**
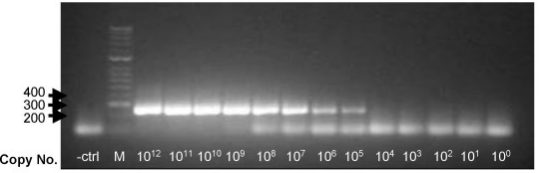
**Sensitivity of the one-step RT-PCR assay**. Example of amplification results of ten-fold serial dilutions of *in-vitro *transcribed RNA H10N7 subtype (refer to Table 2 for strain name). A band of approximately 253 bp was clearly visible with 40 femtogram of starting RNA (equivalent to 10^5 ^copies). M, 100 bp DNA Hyperladder II (Bioline); and negative control (- ctrl: water instead of template).

Ten-fold serial dilutions of *in-vitro *transcribed N1, N7 and N8 cultured RNA sample were prepared from 1 × 10^12 ^to 1.8 × 10^0 ^copy number. For each of the virus subtypes tested, PCR products were visualized by Ethidium Bromide staining on a 1.5% w/v agarose gel (see Figure 5). In testing of dilutions of *in-vitro *transcribed RNA a band of approximately 253 bp was clearly visible with 40 femtogram of starting RNA for N1 and N7 subtypes using 36 cycles. For cultured virus a band of the expected size was visible in the range 10^3 ^(H5N1, 43 cycles) to 10^4 ^(H3N8, 36 cycles) copies. In all cases where bands were visible in agarose gel there was sufficient material for direct sequencing.

## Discussion

Several strategies of RT-PCR have been used to type and subtype influenza A viruses based on different gene segments, such as the Matrix gene [19,26-28], HA gene [5,12,21,28,29], and a few using NA gene [5,21,23,24]. The assays available for the NA gene segment employ subtype-specific primers, which require a number of different, or nested [24] reactions to determine each subtype and may not cover all of the 9 NA subtypes. We are the first to design a primer set for a one-step RT-PCR assay of multiple influenza A viruses that can amplify all 9 NA subtypes simultaneously from a range of host species and from different geographical locations. In our hands, the primers we have designed produced cleaner bands than those obtained using primers described in [24], when applied to similar samples (cultured virus).

An advantage of subtyping influenza A virus by RT-PCR followed by sequencing is the saving of time. The traditional approach for NA subtyping is through NA inhibition assay, sometimes followed by confirmatory PCR for inconclusive results. The NA inhibition assay requires viral culture, and subtyping is obtained within 1–2 weeks. We circumvent this by going directly from extraction of viral RNA from the sample, performing a one-step RT-PCR assay, followed by sequencing and BLAST analysis, thus shortening the time to 2–3 days for NA subtyping.

Another advantage of subtyping the virus by RT-PCR is that sequence analysis of the PCR product, in addition to allowing accurate NA subtyping, could provide important epidemiological information on the origin of the identified influenza virus. This information cannot be provided by NA inhibition assay. In addition, the fragment generated through our RT-PCR can be interrogated for the presence of one of the mutations conferring resistance to oseltamivir, which is crucial for initiating an appropriate treatment and management of outbreaks.

There was one discrepancy between NA types obtained by neuraminidase inhibition test and our one step RT-PCR. Of the four putative N1 samples (three cultured viruses and one clinical sample), typed by Neuraminidase inhibition test, three were confirmed as N1 by our method, but one (the clinical sample) was typed as N2 by our assay. Additional N1 clinical samples will be tested to confirm if there is a systematic misclassification by the neuraminidase inhibition test. In three samples, high quality sequence was not obtainable from the amplified products. One of these samples had visible discoloration which could indicate the presence of compounds that may interfere with the sequencing process. One of the panels of N6 viruses (H13N6) failed to amplify. The Genbank entry for that particular virus does not show mutations in the primer annealing site, so the failure to amplify is not likely to result from sequence drift on the primer site. Nine other viruses of subtype N6 were successfully amplified.

When interpreting clinical NPA amplification results, the percentage obtained (68%) provides support that our assay is likely to be suitable for use for clinical samples. RNA quality was an issue because the clinical samples used, had been stored for a year at -80°C, and freeze-thawed twice prior to running our assay. RNA extracted from fresh clinical samples will be sought in order to determine the positive and negative agreement with other diagnostic methods at multiple testing sites.

## Conclusion

Our data indicates that, compared to Neuraminidase inhibition testing and other RT-PCRs, the newly designed one-step RT-PCR assay offers a faster, accurate and specific tool for the subtyping all 9 NA subtypes of influenza A viruses from a range of Mammalian and Avian species. The sequence information obtained can be helpful in determining the origin of the influenza virus and can be interrogated for the presence of mutations conferring resistance to antiviral drugs. The prompt availability of this information is important for initiating an appropriate treatment and for the tracing and management of outbreaks.

## Methods

### Design of oligonucleotides

#### Neuraminidase (NA) primers design

NA RT-PCR primers were designed based on sequence information obtained from the NCBI Influenza Virus Resource Database (IVRD) [30]. A selection of 1,101 full-length NA sequences of the 9 subtypes, of a range of host species and from different geographical locations were retrieved and aligned using Biological Sequence Alignment editor software (BioEdit, version 7.09, CA, US) [31]. A tabular summary of the nucleotide composition at each position in the alignment was used for the primer design and the strategy was as follows: all positions in the target region had a GAP ≤ 5 (GAP is the number of viruses for which information is lacking regarding nucleotide composition at a particular position of a nucleic acid alignment), and semi-conserved sequence regions of 20 nucleotides long with a redundancy ≤ 195 were sought. Redundancy was then minimized by inserting inosines at more than 1 site.

#### Bioinformatic analysis of designed NA8F/NA10R primers

NA8F and NA10R primers were aligned against the 3,337 sequences retrieved from the IVRD at the time of analysis. The sequence alignment is presented in sequence logo format [32-34]. The percentage of samples with identical matches at all five 3' terminal bases was calculated for each NA subtype.

### Samples and RNA extraction

#### Animal samples from allantoic fluids

Virus (see Table 2) from the reference collection at the Australian Animal Health Laboratory (AAHL) was grown in embryonated eggs. Samples included Influenza virus A from ducks (n = 9), chickens (n = 6), shearwater (n = 3), gull (n = 3), emu (n = 1), other avian species (n = 7), equine (n = 1) and others (n = 2). Viral RNA was extracted from 100 μL of amniotic fluid sample inactivated by addition of 600 μL of RLT buffer (guanidium denaturant) and 6 μL of 2-mercaptoethanol prior to extraction with the QIAGEN RNeasy extraction kit (QIAGEN, Doncaster, Victoria, Australia). Extraction was undertaken as per manufacturer's instructions. RNA was resuspended in 50 μL of nuclease-free water.

#### Clinical specimens

Sixty-three frozen viral RNA extracts from clinical nasopharyngeal aspirate (NPA) specimens were provided as blinded specimens by the Molecular Diagnostic Unit of Pathology Queensland Herston Hospital Campus, Queensland (QLD), Australia, as blind specimens. The samples had been stored at -80°C for one year and thawed twice prior to our study. These specimens, primarily isolated in QLD, were collected from suspect cases of viral respiratory disease between September-October 2006. Patients ranged from 7 weeks to 84 years old with a gender ratio of 58.5% for males and 41.5% females. The blind samples encompass a selection of influenza A viruses, influenza B viruses, and adenovirus (n = 37 influenza A, n = 25 influenza B, n = 1 adenovirus). Viral RNA was extracted from 200 μL of NPA samples using MagNA Pure LC total nucleic acid isolation kit (Roche) and eluted in 100 μL of elution buffer as per manufacturer's protocol. Freshly extracted RNA was initially used by Pathology Queensland to perform sample typing (influenza A/influenza B) using real-time RT-PCR based on the matrix gene specific primers according to Syrmis, *et al*. [27].

In addition, four frozen respiratory syncytial viral RNA (RSV) extracts from clinical NPA specimens were tested. The clinical specimens were from reported cases of viral respiratory disease from Sydney in the period of 2003–2004. Viral RNA was extracted from these samples using High Pure Viral Nucleic Acid kit (Roche) as per manufacturer's protocol.

Previous data (not shown) suggested that RSV clinical RNA samples had genomic DNA contaminants. Therefore, 5 μL of RSV RNA were treated with RNase-Free DNase (Promega) as per manufacturer's protocol, prior to one-step RT-PCR.

### One step-reverse transcription-PCR (RT-PCR)

One-step RT-PCR was performed in 50 μL reaction volume using SuperScript™ III One-Step RT-PCR System with Platinum^® ^*Taq *DNA polymerase kit (Invitrogen, Carlsbad, CA, USA) as per manufacturer's protocol with the following modifications: 4 μmoles/L of each primer (or 1 μmoles/L of each primer for cultured samples) **NA8F-M13 **5'-GTA AAA CGA CGG CCA GT GRA CHC ARG ART CIK MRTG-3'- and **NA10R-M13 **5'-CAG GAA ACA GCT ATG AC CCI IKC CAR TTR TCY CTR CA-3' or **NA8F **5'-GRA CHC ARG ART CIK MRTG-3' and **NA10R **5'-CCI IKC CAR TTR TCY CTR CA-3' and 1 to 2 μL of RNA were added. Thermocycling was performed with the following cycling conditions: 30 min at 46°C and 10 min at 60°C (reverse transcription), 3 min at 94°C (initial denaturation), 8 cycles of step-down PCR consisting of 30 s at 94°C (denaturation), 30 s at 56°C (annealing) – decrease 2°C each cycle until 42°C; and 75s at 68°C (extension). Amplification of the final product was completed for 36 cycles of 30 s at 94°C, 30 s at 43°C, and 75 s at 68°C, with a final extension of 10 min at 68°C for egg cultured and *in-vitro *transcribed RNA samples. For RNA extracted from clinical samples, 43 cycles were used. Reactions were performed in Mastercycler^® ^ep gradient S apparatus (Eppendorf) or MyCycler thermal cycler (Bio-Rad). In the negative control, water for injections BP (Pfizer) or RNase free water (Promega) was used instead of template RNA. The positive control included influenza A/N2 (clinical sample # 12) or influenza A/H3N8/Avian/669/WA/78. Amplicons (10 μL of sample) were visualized by gel electrophoresis on 1.5% agarose containing ethidium bromide. The size of the amplicons generated with M13 tags was approximately 253 bp and amplicons without the M13 tags was approximately 219 bp.

### Sequencing

The 253 bp RT-PCR fragments with M13-tags were direct sequenced as described below. For the 219 bp RT-PCR fragments, indirect sequencing was performed.

#### Direct sequencing

For direct sequencing, whole reaction volumes of the 253 bp amplicon with M13 tags were loaded on 1.5% agarose containing ethidium bromide and gel purified using QIAquick Gel Extraction Kit (QIAGEN) as per manufacturer's instructions. Sequencing reactions using M13 sequencing primers were completed at AAHL or at the Australian Genome Research Facility, AGRF [35], using an automatic sequencer AB3730xl (Applied Biosystems, US). Results were analyzed and influenza virus subtypes were determined by BLAST analysis [36].

#### Indirect sequencing

For indirect sequencing, amplicons (10 μL of sample) of the 219 bp one-step RT-PCR product were initially visualized by gel electrophoresis on 1.5% agarose containing ethidium bromide; and then, whole reaction volumes were loaded and gel purified using QIAquick Gel Extraction Kit (QIAGEN) as per manufacturers' instructions. A PCR was carried out to produce the 253 bp fragment with M13 tags. The PCR was performed using *Taq *DNA polymerase, recombinant kit (Invitrogen) with the following modifications: 4 μmoles/L of each primer NA8F-M13 and NA10R-M13 were used, and 6 ng of gel-cleaned cDNA was used as template. Thermocycling was performed with the following cycling conditions: 3 min at 94°C (initial denaturation), 8 cycles of step-down PCR consisting of 30 s at 94°C (denaturation), 30 s at 56°C then decrease 2°C each cycle until 42°C; 75s at 68°C (extension), followed by 30 cycles of 30 s at 94°C, 30 s at 43°C, 75 s at 72°C, with a final extension of 10 min at 72°C. Reactions were performed in Mastercycler^® ^ep gradient S apparatus (Eppendorf). In the negative control, water for injection BP (Pfizer) was used instead of template RNA. Positive controls included influenza A (N2) (samples # 65, 8 or 12). Amplicons (10 μL of sample) were visualized by gel electrophoresis on 1.5% agarose containing ethidium bromide. Sequencing was performed the same way as described for the direct sequencing method. Results were analyzed and influenza virus subtypes were determined by BLAST analysis [36].

### Sensitivity of the one-step RT-PCR assay

To determine the analytical sensitivity of the one-step RT-PCR using NA8F-M13 and NA10R-M13 primers, ten-fold serial dilutions of *in-vitro *transcribed RNA of the NA fragment amplified by our primers of egg-cultured sample A/Chicken/Cambodia/1A/04/H5N1 (shown in Table 2) were made down to 10 copies/μL transcribed RNA. The concentration of the transcribed RNA (ng/μL) was quantified using the Nanodrop^® ^ND-1000 UV-Vis spectrophotometer (Nanodrop Technologies). Conversion of ng/μL of single stranded RNA to pmol/μL was performed using the following mathematical formula: pmol/μL = ng/μL (of ssRNA) × (1 μg/1000 ng) × (10^6 ^pg/1 μg) × (1 pmol/340 pg) × (1/N); where N = 324 bp, the number of bases of the RNA transcript, and 340 pg/pmol is the average molecular weight of a ribonucleotide. The copy number/μL transcribed RNA was calculated as follows: copy number/μL RNA transcript = (RNA in mol/μL) × (Avogrado constant, 6.023 × 10^23 ^molecules/mol) [37]. Two μL of undiluted RNA stock was used as a positive control, and two μL of each serial dilution was used for the one-step RT-PCR. Amplicons (10 μL/sample) were visualized by gel electrophoresis on 1.5% agarose containing ethidium bromide.

## Abbreviations

AAHL: Australian animal health laboratory; AGRF: Australian genome research facility; bp: Base pair; GAP: Number of occurrences which lack nucleotide information at a determined position of a nucleic acid alignment; HA: Hemagglutinin; IVRD: Influenza virus resource database; M1: Matrix protein; NA: Neuraminidase; N/A: Sample was not assayed with the corresponding test; NCBI: National center for biotechnology information; NP: Nucleoprotein; NPA: Nasopharyngeal aspirate; NSA: RT-PCR positive but no subtype available; NVI: No virus isolated; PCR: Polymerase chain reaction; QLD: Queensland; RLT buffer: RNeasy lysis buffer provided by QIAGEN; RSV: Respiratory syncytial virus; RT-PCR: Reverse transcription PCR; RRT-PCR: Real time reverse transcription PCR.

## Competing interests

The authors VB, EV, WC, CB, and HGH declare that they have no competing interests. ACA, and RL are receiving salary from Biochip Innovations Pty Ltd. MEGB received salary from Biochip Innovations Pty Ltd. RB holds shares in Biochip Innovations Pty Ltd. ACA, RL, and RB are inventors on patent # WO/2008/000023 held by BioChip Innovations Pty Ltd that relates to the content of the manuscript. BioChip Innovations Pty Ltd is financing the processing charge of this manuscript.

## Authors' contributions

ACA designed the primers and participated in the design of the study with assistance from RB, planned and performed the experiments at University of Queensland and drafted the manuscript. MEGB participated in sensitivity assays and edited the manuscript. VB performed experiments to evaluate the one-step RT-PCR assay for N subtyping on cultured virus samples and contributed to editing the manuscript. RL participated in the extraction of RNA and edited the manuscript along with EV, WC, and CB. EV was also involved with virus culture, RNA extraction and RT-PCR validation. WC and CB provided clinical samples, results of neuraminidase inhibition assays and haemagglutinin subtyping and real-time PCR for influenza A/B. HGH instigated the evaluation of the one-step RT-PCR assay and sequencing for N subtyping of influenza at AAHL, helped to draft and edit the manuscript, and participated in the overview of the study. RB conceived of the study, and participated in its design and coordination, helped to draft the manuscript, and participated actively in the overview of the study. All authors read and approved the final manuscript.
